# Asymptomatic *Candida glabrata* urinary tract infection in an immunocompetent young female: A case report

**DOI:** 10.1097/MD.0000000000033798

**Published:** 2023-05-17

**Authors:** Ruixin Deng, Xingye Meng, Ruoyu Li, Aiping Wang, Yinggai Song

**Affiliations:** a Department of Dermatology and Venerology, Peking University First Hospital, Beijing, China; b National Clinical Research Center for Skin and Immune Diseases, Beijing, China; c Research Center for Medical Mycology, Peking University, Beijing, China; d Beijing Key Laboratory of Molecular Diagnosis on Dermatoses, Beijing, China.

**Keywords:** antibiotics, *Candida glabrata*, *CARD9* mutation, multidrug resistance, urinary tract infection

## Abstract

Fungal urinary tract infections (UTIs) are becoming increasingly common in hospitalized patients and *Candida* species are the most prevalent organisms. However, recurrent candiduria in young healthy outpatients is rare thus require further examination to find the etiologic factors.

**Case presentation:**

We described a case of recurrent asymptomatic c caused by azole-resistant *C. glabrata* in a healthy young female who only had previous use of antibiotics without other risk factors. However, after removal of the predisposing factor and the use of sensitive antifungal agents, the patient’s urine cultures remained positive. This phenomenon indicated to us that the patient might have an immune-related genetic deficiency. We found a novel caspase-associated recruitment domain-containing protein 9 (CARD9) gene mutation (c.808-11G > T) which might be the cause of recurrent asymptomatic candiduria in this immune-competent young female without any underlying diseases.

**Conclusions:**

We report a case of recurrent asymptomatic candiduria caused by azole-resistant *Candida glabrata* in a young healthy female with a novel *CARD9* mutation. A functional study of this mutation should be performed in the future to determine its effect on asymptomatic fungal UTIs.

## 1. Introduction

Urinary tract infections (UTIs) are one of the most common infections in both communities and hospitals,^[1]^ and *Escherichia coli* infections account for the largest proportion of UTIs in China.^[2]^ In addition to bacterial UTI, fungal UTI has become an increasingly more common nosocomial infection, and *Candida* species are the most prevalent organisms.^[3]^ Studies have shown that >50% of urinary Candida isolates are nonalbicans species, although *C. albicans* is the most commonly observed species in urine culture.^[4]^ Among them, *C. glabrata,* in particular, is substantially more resistant to antifungals, allowing it to quickly evolve resistance to treatment.^[5]^ As antifungal drugs are being used more widely, *C. glabrata* isolates that are resistant to fluconazole and echinocandins are being found more often in clinical settings, which is a growing threat to public health.^[6]^ To prevent the spread of drug-resistant strains of *Candidas* spp. and to minimize deaths from these infections, it is crucial to practice antifungal management and reduce the inappropriate use of antifungals. Since most patients with candiduria are asymptomatic,^[7]^ the decision to use antifungal agents and how to determine the optimal therapeutic strategy remain challenging.

Candiduria is commonly observed in hospitalized patients, who may be predisposed to various risk factors. However, recurrent candiduria in young healthy outpatients is rare, and thus further examination is required to identify the etiologic factors. Evidence suggests that a lack of caspase-associated recruitment domain-containing protein 9 (CARD9) may contribute to chronic Candida infections.^[8]^ However, CARD9-deficient patients have not been observed to have asymptomatic candiduria. Here, we describe a CARD9 gene variation in a young woman with azole-resistant *C. glabrata* that causes asymptomatic candiduria.

## 2. Case report

**Figure 1.**
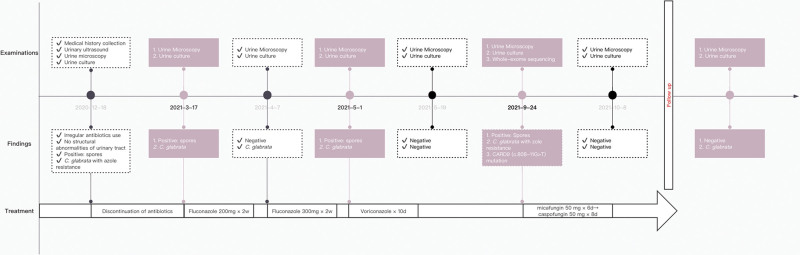
Illustration of the case progression.

**Figure 2.**
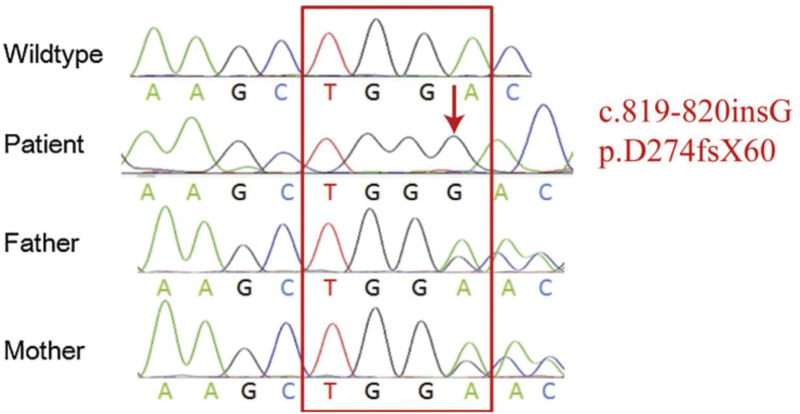
CARD9 mutation of the patient. CARD9 = caspase-associated recruitment domain-containing protein 9.

A 21-year-old woman with a history of inconsistently taking antibiotics complained of turbid, white-striped urine for the previous 2 months (2020-12-18). She did not experience dysuria, increased frequency or urgency of urination, fever or flank pain. Her vital signs and physical exam results were essentially unremarkable except dermatographism. Her parents had no relevant medical history. The ultrasound revealed no obvious abnormalities of the kidneys, ureters or bladder. Urine microscopy revealed spores, and urine culture was positive for *C. glabrata* (more than 10 × 10^6^ colony-forming units/L), which was resistant to fluconazole (minimal inhibitory concentration [MIC] > 256 µg/mL), voriconazole (MIC > 8 µg/mL), itraconazole (MIC > 16 µg/mL), and posaconazole (MIC > 8 µg/mL) but sensitive to amphotericin B (MIC 1 µg/mL), micafungin (MIC 0.015 µg/mL), caspofungin (MIC 0.12 µg/mL), 6-anifengin (MIC 0.03 µg/mL) and 5-flucytosine (MIC < 0.06 µg/mL). The patient received no treatment except for discontinuation of antibiotic use according to guidelines. However, subsequent urine cultures were still positive. Therefore, a 2-week course of oral fluconazole (200 mg per day) was administered (2021-3-17). Afterward, the patient had less cloudy urine and fewer white strips; the urine microscopy results became negative, but the urine culture remained positive for *C. glabrata* (4.7–4.21). Despite treatment with another 2-week course of oral fluconazole (300 mg/d), the urine cultures were still positive for *C. glabrata* (Fig. 1). The patient then received a 10-day course of oral voriconazole, which was withdrawn due to adverse reactions, including nausea, vomiting and photophobia. Although a negative urine fungal culture was obtained after treatment (5–19), follow-up urine cultures were again positive for *C. glabrata* with similar susceptibility to antifungal drugs (6.9, 7.14, 9.15, 9.23). To further clarify the etiology, blood whole-exome sequencing was performed. *CARD9* mutation (c.808-11G > T) was found in the patient (Fig. 2). During hospitalization, intravenous micafungin 50 mg was given every day for 6 days (9.24–9.29), which was followed by caspofungin 50 mg every day for 8 days (9.30–10.8). A urine culture performed after the last caspofungin dose revealed no significant growth. However, the patient experienced further recurrence of asymptomatic candiduria during a 6-month follow-up, during which urine microscopy results remained negative, whereas the urine cultures were persistently positive (2022-1.12, 3.18, 4.8).

## 3. Discussion and conclusions

One of the most often diagnosed infections in both medical facilities and the general public is urinary tract infection. The occurrence of nosocomial infections caused by *Candida* spp. has grown considerably during the last decade, contributing to major issues of public health.^[9]^ The presence of diabetes mellitus, advanced age, urinary tract device implantation, concurrent bacteriuria, renal transplantation, urinary tract abnormalities, intensive care unit hospitalization, and antibiotic exposure have been identified as major risk factors. Community-acquired candiduria was found to be more common in young, pregnant, and bedridden patients.^[10]^ In our study, the patient was a healthy young female without immunosuppression, pregnancy, bedridden status, hospitalization, or abnormalities of the kidneys, ureters or bladder; the only risk factor was the previous use of antibiotics. *Candida* infection could be induced by the use of broad-spectrum antibiotics since it may deplete the flora of the digestive tract and the genital tract.^[7]^ However, after removal of the predisposing factor, the patient’s urine cultures remained positive. This finding indicated to us that the patient might have an immune-related genetic deficiency. CARD9, an adapter protein, is essential for host defense against fungal infections because it connects C-type lectin receptors to nuclear factor kB-driven gene expression.^[11]^ CARD9 deficiency was initially reported in a multiplex consanguineous family in Iran in which 6 of 7 members had chronic mucocutaneous candidiasis.^[12]^ To date, over twenty-two distinct *CARD9* mutations have been discovered, including both homozygous and compound heterozygous mutations.^[13]^ Our study revealed a novel *CARD9* mutation that might be the cause of recurrent asymptomatic candiduria in this immunocompetent young female who did not have any underlying diseases. A functional study of this mutation should be performed in the future to determine its effect on asymptomatic fungal UTIs.

Most cases of candiduria (50–70%) are caused by *Candida albicans,* followed by *Candida glabrata* (20%) and *Candida tropicalis* (10%). The extensive use of immunosuppressive drugs and azole treatment has led to an increase in the occurrence of nonalbicans *Candida* candiduria and notably that caused by *C. glabrata.* Surveillance population surveys have shown rising frequencies of fluconazole resistance in nonalbicans species. In a Danish surveillance study, an increase in azole resistance was observed in 2012–2015; the rate of isolates being susceptible to azoles rose to 60.6% compared with 55.2% and 58.5% in 2008–2011 and 2004–2007, respectively. In that study, *C. glabrata* accounted for 9.1% of isolates and was largely responsible for the high rates of nonsusceptibility.^[14]^ SENTRY records show that although the incidence of fluconazole resistance among *C. albicans* isolates continues to be modest (0.3%), it is rather high among *C. glabrata* isolates (8.1%).^[15]^ In North America, 10.6% of *C. glabrata* isolates are resistant; this rate is followed by that in the Asia-Pacific region at 6.8% and that in Europe at 4.9%.^[16]^ Since *C. glabrata* isolates cause not only asymptomatic candiduria but also invasive candidiasis,^[17]^ rising antifungal resistance would lead to a limited number of treatment options, resulting in higher mortality. Therefore, rational application of antifungal agents is critical in treating Candida infection and reducing resistance emergence.

Despite the availability of published recommendations, the standardized treatment of candiduria remains controversial due to the challenges inherent in identifying the clinical implications and importance of yeast in urine. In most cases involving adult patients, candiduria implies colonization or contamination of the material cultured rather than infection,^[18]^ making it essential to differentiate UTIs from other clinical conditions. The presence or absence of *Candida* in fresh urine samples enables easy differentiation between contamination and colonization or UTI.^[19]^ Patients with the persistent presence of *Candida* species in the urine can be divided into 4 clinical categories: patients with asymptomatic candiduria (predisposed inpatients); patients with asymptomatic candiduria (previously healthy or predisposed outpatients); patients with symptomatic candiduria (UTI) and clinically unstable patients with candiduria.^[20]^ Asymptomatic patients who have *Candida* species in their urine do not need to be treated with antifungal medication unless they are neutropenic, have a very low weight, or are having urologic surgery.^[21]^ Therefore, asymptomatic candiduria may often be remedied without the use of antifungal medication if certain clinical circumstances are addressed or risk factors are eliminated.^[18]^ Our patient’s sole recognized risk factor was antibiotic misuse. The premature cessation of antimicrobials has been associated with a decrease in the levels of nonbacterial emerging microbes.^[20]^ Despite this, it seemed that the presence of Candida in the urine in our patient could not be eradicated simply by stopping antibiotic treatment. Hence, antifungal therapy was then administered according to the patients’ strong desire.

According to guidelines, oral fluconazole is the first-line therapy for UTIs caused by *Candida*.^[4]^ Due to its high levels in urine, fluconazole is effective against infections of the upper and lower tracts.^[4]^ For fluconazole-resistant *C. glabrata*, amphotericin B deoxycholate is recommended.^[22]^ Moreover, lipid formulations cannot achieve acceptable concentrations in urine and have been reported to fail when used to treat UTIs caused by *Candida*; thus, they are not advised for use in treating UTIs.^[23]^ Flucytosine, an agent that reaches high concentrations in the urine and is effective against a variety of isolates of *C. glabrata*, is also helpful in treating fluconazole-resistant UTIs caused by *C. glabrata*.^[24]^ After the failure of azole treatments, echinocandins were finally chosen as the second-line therapy for azole-resistant candiduria in our study considering the susceptibility of the fungus and the adverse effects and accessibility of the antifungal agents. Echinocandins are an emerging family of antifungal drugs with broad-spectrum fungicidal action against *Candida* spp., although they are rarely explored in the treatment of UTIs because they do not achieve therapeutically relevant concentrations in urine.^[24]^ Recently, there has been increasing evidence indicating that echinocandins may be useful in the treatment of candiduria caused by fluconazole-resistant *C. glabrata*.^[25]^ Unfortunately, the patient in our study experienced recurrence of candiduria after echinocandins treatment. For patients with resistant infections in whom the predisposing factors have been addressed, more research is required to determine the necessity and benefit of further treatment for recurrent asymptomatic candiduria that is refractory to multiantifungal drugs.

There are some limitations to our study. First, a functional study was not performed on the discovered mutation. In addition, the mechanism by which *CARD9* mutation generates recurrent asymptomatic candiduria could not be revealed. Moreover, we did not treat the patient as recommended by the guidelines; hence, we were unable to observe the efficacy of amphotericin B and flucytosine on recurrent asymptomatic candiduria.

In conclusion, we reported a case of recurrent asymptomatic candiduria caused by azole-resistant *C. glabrata* in a young healthy female with a novel *CARD9* mutation. A history of antibiotic use should be carefully evaluated in outpatients who do not have other underlying diseases. Although most asymptomatic candiduria infections require no antifungal treatment and resolve within weeks to months, patients might experience persistent candiduria even after the risk factors are addressed. In this condition, whole-exome sequencing is necessary to further determine the etiology. Whether to initiate antifungal treatment depends on the patients’ requirements, the susceptibility of the fungus and the adverse effects and accessibility of antifungal agents.

## Author contributions

**Conceptualization:** Ruixin Deng, Xingye Meng, Yinggai Song.

**Investigation:** Yinggai Song.

**Supervision:** Ruoyu Li, Aiping Wang, Yinggai Song.

**Writing – original draft:** Ruixin Deng, Xingye Meng.

**Writing – review & editing:** Ruixin Deng, Xingye Meng, Ruoyu Li, Aiping Wang, Yinggai Song.
